# *Wolbachia-*driven selective sweep in a range expanding insect species

**DOI:** 10.1186/s12862-021-01906-6

**Published:** 2021-09-25

**Authors:** Junchen Deng, Giacomo Assandri, Pallavi Chauhan, Ryo Futahashi, Andrea Galimberti, Bengt Hansson, Lesley T. Lancaster, Yuma Takahashi, Erik I. Svensson, Anne Duplouy

**Affiliations:** 1 Department of Biology, Lund University, Sölvegatan 37, 223 62 Lund, Sweden; 2 Institute of Environmental Sciences, Jagiellonian University in Kraków, Gronostajowa 7, 30-387 Kraków, Poland; 3 Area per l’Avifauna Migratrice, Istituto Superiore per la Protezione e la Ricerca Ambientale (ISPA), Via Ca’ Fornacetta 9, 40064 Ozzano Emilia, BO Italy; 4 Bioproduction Research Institute, National Institute of Advance Industrial Science and Technology (AIST), Trukuba, Ibaraki 305-8566 Japan; 5 Department of Biotechnology and Bioscience, University of Milano-Bicocca, Piazza della Scienza 2, 20126 Milan, Italy; 6 School of Biological Sciences, University of Aberdeen, Aberdeen, AB24 2TZ UK; 7 Graduate School of Science, Chiba University, Chiba, Japan; 8 Insect Symbiosis Ecology and Evolution Lab, Organismal and Evolutionary Biology Research Program, The University of Helsinki, Viikinkaari 1, 00014 Helsinki, Finland

**Keywords:** Endosymbiosis, Phylogeography, Damselfly, Mitochondria, Genetic diversity

## Abstract

**Background:**

Evolutionary processes can cause strong spatial genetic signatures, such as local loss of genetic diversity, or conflicting histories from mitochondrial versus nuclear markers. Investigating these genetic patterns is important, as they may reveal obscured processes and players. The maternally inherited bacterium *Wolbachia* is among the most widespread symbionts in insects. *Wolbachia* typically spreads within host species by conferring direct fitness benefits, and/or by manipulating its host reproduction to favour infected over uninfected females. Under sufficient selective advantage, the mitochondrial haplotype associated with the favoured maternally-inherited symbiotic strains will spread (i.e. hitchhike), resulting in low mitochondrial genetic variation across the host species range.

**Method:**

The common bluetail damselfly (*Ischnura elegans:* van der Linden, 1820) has recently emerged as a model organism for genetics and genomic signatures of range expansion during climate change. Although there is accumulating data on the consequences of such expansion on the genetics of *I. elegans*, no study has screened for *Wolbachia* in the damselfly genus *Ischnura*. Here, we present the biogeographic variation in *Wolbachia* prevalence and penetrance across Europe and Japan (including samples from 17 populations), and from close relatives in the Mediterranean area (i.e. *I. genei*: Rambur, 1842; and *I. saharensis*: Aguesse, 1958).

**Results:**

Our data reveal (a) multiple *Wolbachia*-strains, (b) potential transfer of the symbiont through hybridization, (c) higher infection rates at higher latitudes, and (d) reduced mitochondrial diversity in the north-west populations, indicative of hitchhiking associated with the selective sweep of the most common strain. We found low mitochondrial haplotype diversity in the *Wolbachia*-infected north-western European populations (Sweden, Scotland, the Netherlands, Belgium, France and Italy) of *I. elegans*, and, conversely, higher mitochondrial diversity in populations with low penetrance of *Wolbachia* (Ukraine, Greece, Montenegro and Cyprus). The timing of the selective sweep associated with infected lineages was estimated between 20,000 and 44,000 years before present, which is consistent with the end of the last glacial period about 20,000 years.

**Conclusions:**

Our findings provide an example of how endosymbiont infections can shape spatial variation in their host evolutionary genetics during postglacial expansion. These results also challenge population genetic studies that do not consider the prevalence of symbionts in many insects, which we show can impact geographic patterns of mitochondrial genetic diversity.

## Background

Range expansion studies have uncovered waves of demographic expansion in many species by comparing the genetic diversity of the initial source to that of the edge populations. Often, but not always (e.g. [1]), range expansions lead to reduced genetic diversity and stronger genetic differentiation at a species range limits compared to the source populations. These patterns are often due to the action of drift during rapid demographic spatial expansion and colonization [2, 3]. Under these conditions, certain alleles and genotypes have been shown to spread in the newly colonized regions due to allele surfing [4], or due to selection for local adaptation in novel environments at the range limits [1, 5–8]. Hidden processes and players may however confound these patterns, and challenge our full understanding of the evolutionary histories and genetic diversity of source and edge populations. Infections with maternally inherited symbionts, for example, can cause loss of genetic diversity across entire populations because of differential selection pressures on the infected versus uninfected host lineages, a process that may masquerade as selection or drift. The selective sweep of maternally inherited symbionts can lead to the hitchhiking of certain host haplotypes, which may not themselves be the major targets of selection [9–12].

The maternally inherited symbiotic bacteria *Wolbachia* can be found in up to half of all arthropod species [13, 14]. These bacteria are selfish passengers known for manipulating their host reproductive system via, for example, inducing the killing or feminization of the male progeny [15, 16], and the overproduction of daughters from unfertilized eggs (thelytoky [17]), or causing incompatibility between males and females of different infection status [18]. Some *Wolbachia* strains have also been shown to provide nutrients essential to the survival of their hosts [19], to protect against natural enemies [20–22], or to influence their host behaviours in ways that enhance fitness (e.g. mating rate [23], lekking [24], host choice [25], and more—see review [26]). These phenotypes have been selected as they improve the fitness of infected hosts over their uninfected counterparts, and therefore provide efficient means for the spread of the symbiont through generations, which can affect the inheritance pattern of the host DNA [27]. Rapid spread of maternally transmitted *Wolbachia* across populations and within species can lead to hitchhiking of the co-inherited mitochondrial haplotypes, increasing their frequencies in the host population. As a result, the overall mitochondrial diversity has been shown to decrease within infected populations [28].

In addition to potentially biasing population genetic signals of selection and drift, hidden *Wolbachia* infections could mislead, or challenge, species identification [29] and genetic and phylogenetic inferences based on the mitochondrion, as infection with such symbiont can lead to mito-nuclear discordance or affect diversification processes [30]. Integrating data about the infection-status of species as part of the routine protocol of genetic and phylogenetic studies, could for example inform on the obscure mitochondrial history of closely related species and hybrids [31].

The insect order Odonata (dragonflies and damselflies) includes approximately 6400 species belonging to 32 families [32]. Within these, the damselfly genus *Ischnura* (bluetails/forktails; Zygoptera: Coenagrionidae) is broadly distributed in both the Old and the New Worlds. Members of this damselfly genus and other odonates have been shown to readily undergo range shifts and expansion in response to climate change [33]. In the clade containing the common bluetail damselfly (*Ischnura elegans*), hybridization and introgression have been reported between *I. elegans* and the island bluetail (*I. genei*), the Sahara bluetail (*I. saharensis*) [34], and the Iberian bluetail (*I. graellsii*) [35], under secondary sympatry following recent range shifts [34–36]. Other studies have shown significant adaptive allele frequency changes along the northward range expansion gradient in *I. elegans*, consistent with selection caused by diverse novel environmental conditions, such as temperature, precipitation and wind speed [1]. Several *Ischnura* species, including *I. elegans*, often have heritable, female-limited colour polymorphisms that include a heritable male-mimic (androchrome; blue females) and one to two other morphs (red or green) [37–40]. Recent studies have shown that these morphs vary in their resistance and tolerance to parasitism by water mites [41], and in various aspects of thermal performance at the northward edge of their range [42, 43].

Although patterns and consequences of *Wolbachia* host interactions have been studied extensively in other insect groups (e.g. Hymenoptera [44]; Lepidoptera [10]; Diptera [45]), the recent studies by Thipaksorn et al. [46], Salunkhe et al. [47] and Lorenzo-Carballa et al. [48] possibly represent the only three systematic studies on *Wolbachia* infection in Odonata, and no previously published study has focused on *Wolbachia* in the genus *Ischnura.* Here, we investigated *Wolbachia* strain diversity in three *Ischnura* species (*I. elegans*, *I. genei*, and *I. saharensis*) and provide a first low-coverage assembly of the most-commonly found strain infecting *I. elegans* in Europe. By sequencing four mitochondrial markers (2375 bp) and one nuclear marker (512 bp), we quantified host genetic diversity, and (I) tested whether any *Wolbachia*-induced selective sweep that might have reduced genetic diversity across the *I. elegans* species range, and (II) looked for evidence of horizontal transfer of the symbiont between the three *Ischnura* species. Finally, by comparing the *Wolbachia* infection status of the three female colour morphs and the males of *I. elegans*, we tested whether there was any indication that the morphs differ in their infection status, just as they do in terms of ectoparasitic water mite infection [41]. This study thus reveals previously hidden players in the ecology and evolution of the range expanding species *I. elegans*, and three of its relatives*.*

## Methods

### Samples collection

**Fig. 1:**
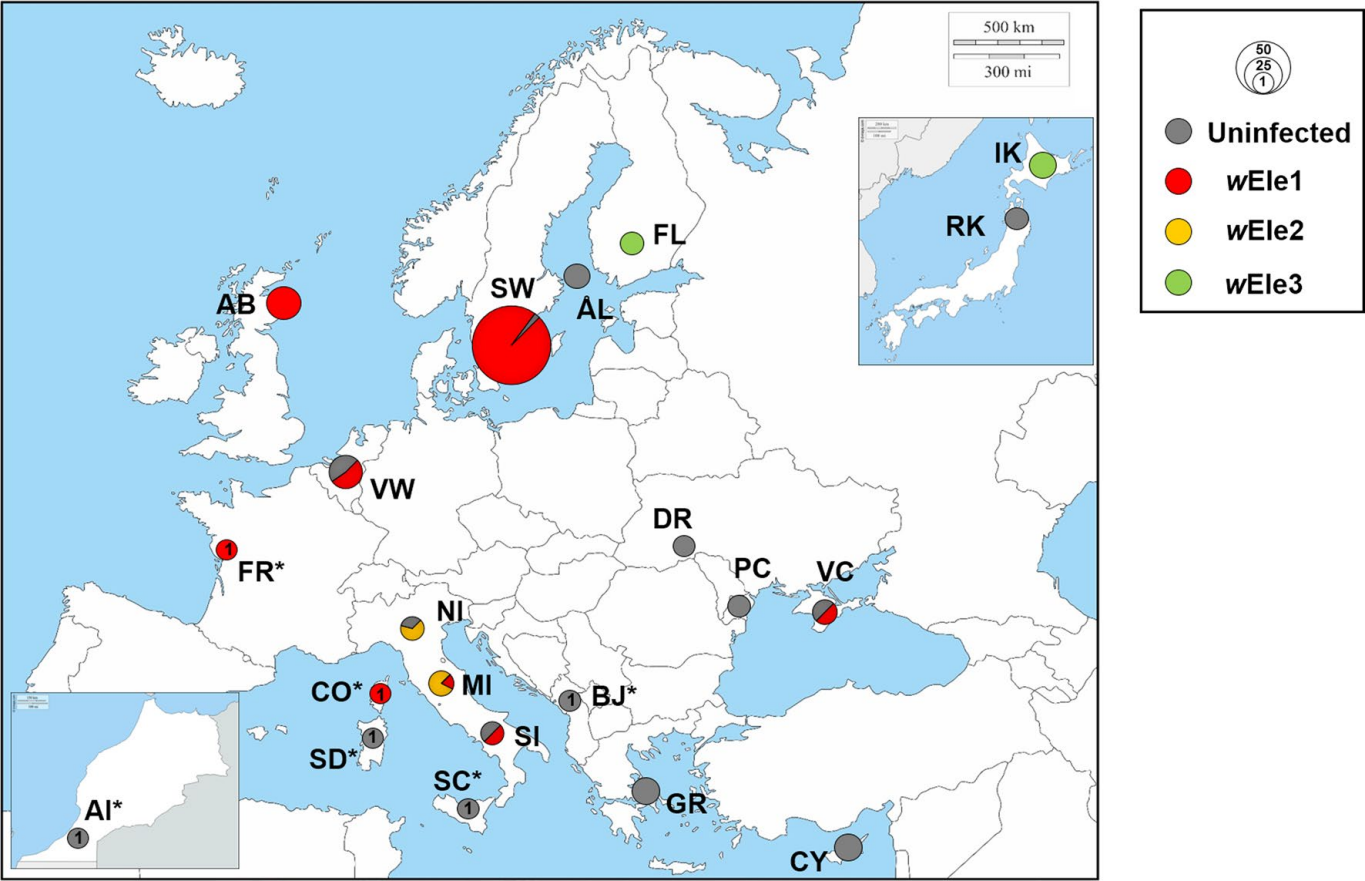
*Wolbachia* strain diversity and penetrance from 17 populations across the geographical range of the damselfly *Ischnura elegans*. The top right window shows the data from the two populations in Japan. The sample in Morocco [AI] (bottom left) is from the species *I. saharensis*, while the samples from [CO], [SC] and [SD] are *I. genei*. Size of each pie chart is proportional to the number of individuals included in the study. ‘*’: *Wolbachia* infection rates in these populations are only based from the screening of one unique individual. Thus, for these populations we might be providing an over or under-estimations of the true local prevalence of the bacterium. Maps were modified from maps freely available here: Europe (https://d-maps.com/carte.php?num_car=2232&lang=en), Japan (https://d-maps.com/carte.php?num_car=354&lang=en), Morocco (https://d-maps.com/carte.php?num_car=1132&lang=en)

*Ischnura* damselflies were collected during the summer of 2015, 2016, 2019 or 2020 depending on their geographical origins. Individuals were caught in the field and stored in 95% ethanol in a − 20 °C freezer until further analysis. Specimens included 87 individuals from seven local populations in South Sweden (15 males and 72 females of all three morphs), and 105 other individuals from twelve other geographic regions, including Finland (Åland islands and mainland), Scotland, France, Cyprus, Greece, Ukraine, Belgium, the Netherlands, Italy, Montenegro and Japan (Table 1). We aimed for a minimum of three specimens per population but also included unique individuals from some regions. The seven Swedish local populations are all located within a few kilometres from each other; thus, samples were grouped under a unique ‘Southwest’ population for the rest of the study. Similarly, samples from Belgium and the Netherlands were grouped as one unique population, denoted as ‘Vinne-Walem’. In contrast, the samples from isolated parts of Italy were separated in three populations based on their relative geographical locations, and denoted either ‘Northern’, ‘Central’, or ‘Southern’ (Fig. 1, Table 1).

**Table 1:**
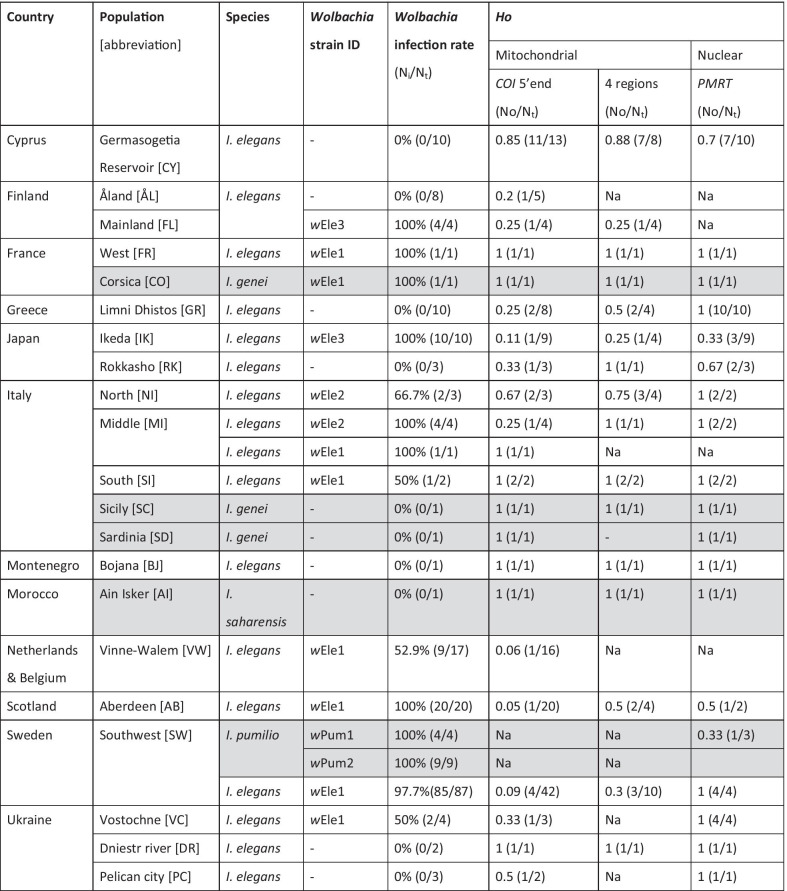
*Wolbachia* strain infection rates and observed haplotype diversity at the mitochondrial (mitotypes based on *COI* 5′end only, or four mitochondrial regions: *COI*, *COIb*, *COIIa*, & *NDI*) and nuclear levels across all populations of four *Ischnura* species

Shaded cells for data on *I. genei*, *I. pumilio*, and *I. saharensis*

*Ho* observed haplotype diversity (as N_H_/N_t_), *N*_*i*_ number of samples infected with *Wolbachia*, *N*_*t*_ total number of samples (screened for *Wolbachia* or genotyped), *No* number of haplotypes chracterized, *Na* failed sequencing

We also included 20 specimens belonging to three other *Ischnura* species in this study:

- Thirteen specimens of *I. pumilio*, collected in 2019 from four Swedish populations. *Ischnura pumilio* co-occurs sympatrically with *I. elegans* in this region but falls in another major phylogenetic clade of the *Ischnura* tree, and is less closely related to *I. elegans* than are the following two species [49].
- Three specimens of *I. genei*, an allopatric species to *I. elegans* endemic of the western Mediterranean region. Two samples of *I. genei* were collected from the two insular populations of Sardinia and Sicily (Italy), and the third sample is from Corsica (France) [50]
- One unique specimen of *I. saharensis* collected from Morocco. The species is also an allopatric species to *I. elegans* and is distributed across North Africa.

### Molecular work

Specimens were dissected in sterile conditions to avoid cross specimen contamination. We extracted the DNA from the abdomen of each damselfly, except for the Japanese samples, for which DNA was extracted from one leg, following the protocol of a Qiagen DNeasy Blood & Tissue Extraction Kit (Cat. #69506, Qiagen, USA). The quality of all DNA extracts was tested by PCR, through the amplification of the 5′-end region (~ 654 bp) of the *cytochrome oxidase I* (*COI*) mitochondrial gene using the primers LCO-1490/HCO-2198 designed by Folmer et al. [51]. Only samples that were positive for the *COI* amplification were included in the following analyses.

All sequences were deposited into the GenBank database (Accession #MZ463094-100; MZ501175-205; MZ508997-9001; MW509059-66; MZ893225-MZ893331). In total, we amplified and sequenced four mitochondrial regions (*COI*, *COIb*, *COIIa* and *NDI*) to test for a selective sweep, one nuclear marker (*PRMT*) to test for population bottlenecks, and two *Wolbachia* genes (*ftsZ* and *wsp*) to characterize strain diversity. Note: an extra *Wolbachia* gene (*fbpa*) was sequenced for few *w*Ele1-infected specimens from Sweden (primers details in Additional file 1: Table S2). Purified PCR products from independent PCRs were sent to Macrogen (Macrogen Europe, Inc.) for single strand direct forward Sanger sequencing. All sequences were manually curated and aligned using Geneious Prime 2020.2.4 (https://www.geneious.com) and AliView 1.26 [52]. Double peaks in the chromatograms were treated as either evidence of contamination (for mtDNA), multiple infections (for *Wolbachia* DNA), polymorphism (for mtDNA and nuclear DNA), or sequencing noise (all). *Wolbachia* and mtDNA sequences showing such patterns were not included in the following analyses, while the analysis of those double peaks in the nuclear locus sequences allowed us to identify heterozygotic and homozygotic specimens at polymorphic sites (see below).

### *Ischnura elegans* nuclear haplotype diversity

To identify diversity at the nuclear level, we isolated 400 bp of the successfully sequenced nuclear gene *PRMT* from 48 specimens (27 uninfected and 21 *Wolbachia*-infected) from 10 populations ([AB], [RK], [IK], [SW], [VC], [MI], [NI], [SI], [CY] and [GR]), with 2–12 specimens per population. However, Italy ([MI], [NI] & [SI]) and Japan ([RK] & [IK]) carry *Wolbachia* strains that are divergent from *w*Ele1, and which may have altered the genetics of those populations in ways that are impossible to fully test due to our small sample size for these populations. Therefore, we did not include these sequences in further analyses. The final sample size was 31 sequences, including 23 *Wolbachia*-uninfected specimens and 8 *Wolbachia*-infected specimens from five populations (Table 1). To test whether the nuclear gene from *Wolbachia*-infected and uninfected damselflies has evolved under neutrality, we performed neutrality tests in DnaSP v6.0 [53] by calculating Tajima’s *D* (Tajima, 1989) and Fu and Li’s *F* [54] metrics, and by estimating nucleotide diversity (π) and haplotype diversity (*Hd*) (Table 2). We also estimated the observed heterozygosity levels at two nuclear polymorphic sites for both the *Wolbachia*-infected and uninfected specimens.

**Table 2:**
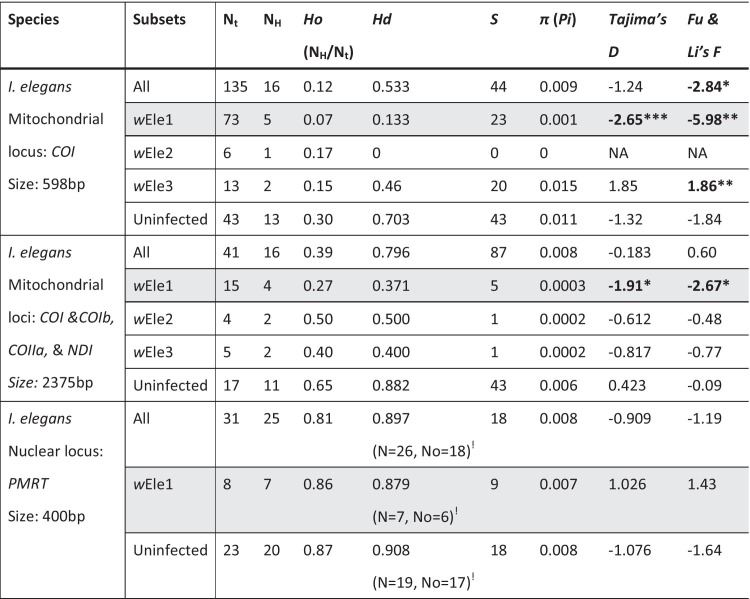
Mitochondrial and nuclear nucleotide diversity estimates, and neutrality tests of *Wolbachia* infected and uninfected *I. elegans* specimens based on the mitochondrial or nuclear loci

*N*_*t*_ number of samples, *N*_*H*_ number of haplotypes, *Ho* observed haplotype diversity (as N_H_/N_t_), *Hd* haplotype diversity (as the probability of two haplotypes to be different), *S* Number of polymorphic sites, *π* nucleotide diversity

*P < 0.05, **p < 0.02, ***P < 0.001. In bold the significant data, and in grey the data for *w*Ele1 infected samples, for visualisation

^!^Sample size and haplotype numbers for the calculations of *Hd*, S, *π*, *Tajima’s D* and Fu & Li’s indexes were slightly different for the nuclear gene, due to an indel in some sequences

### *Wolbachia* and mitochondrial haplotype diversity, phylogenies and haplotype networks

All *Wolbachia* Sanger-sequences were BLAST-ed against the *Wolbachia* PubMLST database (https://pubmlst.org/wolbachia/) [55] to find the corresponding or closest alleles at each locus. Additionally, all five MLST genes (*ftsz*, *gatB*, *hcpA*, *coxA*, and *fbpA*) and the *wsp* gene of the strain *w*Ele1 were also extracted from the whole genome project of a Swedish *I. elegans* [56]. All *Wolbachia* reads were identified and isolated from the raw read data of the *I. elegans* genome project [56]. The *w*Ele1 assembly was built by mapping reads to two previously sequenced *Wolbachia* genomes (*w*Pip [57] and *w*Mel [58]) using bwa mem version 0.7.8 [59]. The properly mapped pairs were extracted using samtools 1.8. The isolated *Wolbachia* paired reads were assembled into a draft genome using spades version 3.9.0 at kmers 21, 33, 55, 77 and 99. The wEle1 draft genome assembly (N_scaffold_ = 893; N50 = 5523 bp; longest scaffold = 53,331 bp, genome size = 1.4 MB) is available as Additional file 2, and the raw reads are available from NCBI under the Bioproject PRJNA575663.

**Fig. 2:**
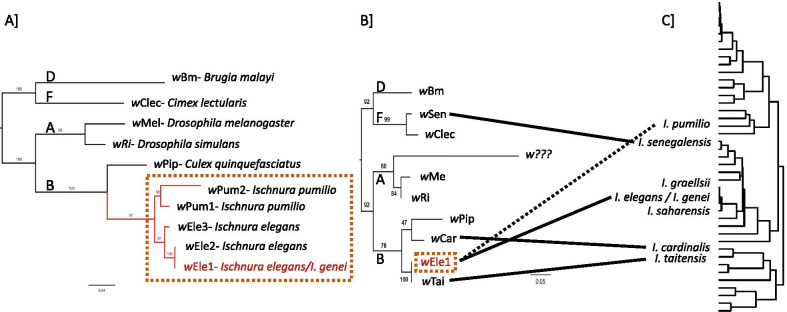
Maximum likelihood tree of (**A**) the *Wolbachia* strains characterized in the present study and based on the concatenated sequences of the *ftsZ* and *wsp* genes, and of (**B**) five *Wolbachia* strains from five *Ischnura* species, including *w*Ele1, based on the *fbpa* gene. **C** Phylogenetic tree of the *Ischnura* genus phylogeny, for comparison, as provided by [70]. In (**B**): the strains marked with red come from *Ischnura* species: *w*Sen [44], ‘*Wolbachia*_*I.* spp.’, *w*Car and *w*Tai [45]. Five additional strains (*w*Bm, *w*Clec, *w*Mel, *w*Ri, *w*Pip) were also included in the trees as references for the different *Wolbachia*-supergroups A, B, D and F. The two *Wolbachia* trees were rooted using the D and F-*Wolbachia* supergroups as outgroups. Bootstrapping was conducted using ‘Ultrafast’ bootstrap method in IQ-Tree with 1000 replicates. Links between the **B** and **C** trees show the lack of concordance between the symbiont and host trees

We concatenated sequences of the *ftsZ* and *wsp* genes from each *Wolbachia* strain characterized in this study, and from five additional strains (*w*Mel, *w*Ri, *w*Clec, *w*Bm, *w*Pip; Isolate id number: 1, 11, 36, 37, 1808 in *Wolbachia* pubMLST database, respectively) previously assigned to the A-, A-, F-, D-, and B-*Wolbachia* supergroups, respectively. The phylogenetic analyses were conducted in IQ-Tree on XSEDE [60] implemented in CIPRES v.3.2 [61], using the genes separately (Additional file 1: Fig. S1) or concatenated (Fig. 2). ‘Model Selection’ [62] was selected to allow for the search of the best model in CIPRES. The partition type was set to allow the two partitions (one for each gene) to have different speeds [63]. The best fit substitution models were decided by running ‘-m TESTNEW’ in IQ-Tree. Bootstrapping was conducted using ‘Ultrafast’ and ‘SH-aLRT’ bootstrap methods (Hoang et al. 2018) in IQ-Tree with 1000 replicates. The ‘TN + F + I’ and ‘TPM3 + F + G4’ models were applied to *ftsZ* and *wsp* genes, respectively, as the best fit models with highest BIC (Bayesian information criterion) scores. All other setting options were left as default. The pairwise genetic distances between *Wolbachia* strains were calculated in MEGA-X [64]. The best phylogenetic trees were visualized in FigTree v.1.4.4. (http://tree.bio.ed.ac.uk/software/figtree/), and rooted using the *w*Bm-D and *w*Clec-F strains as outgroups (Fig. 2).

**Fig. 3:**
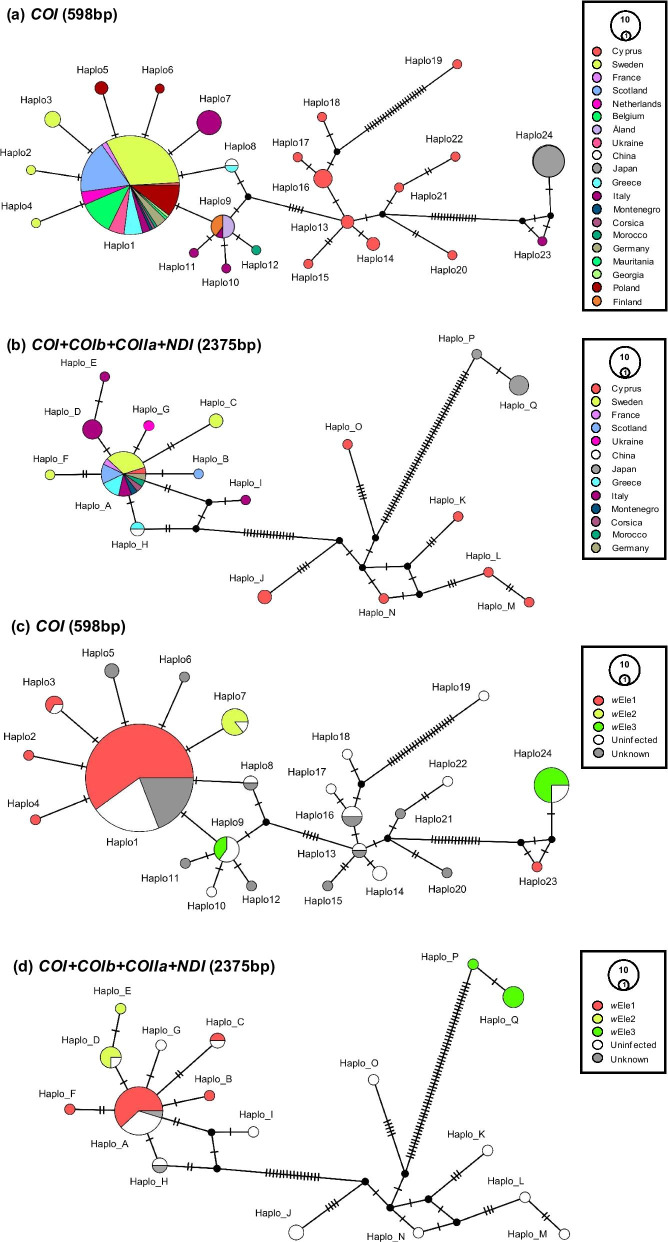
Mitochondrial haplotype networks of *I. elegans* and two closely related species*, I. genei* and *I. saharensis*, based on (**a** and **c**) the mitochondrial *COI* gene only; and (**b** and **d**) all four mitochondrial markers, organised per country (**a** and **b**) or per infection status (**c** and **d**). Each circle represents one unique haplotype, which might be biased by differences in our sampling effort between populations. The size of the circle is proportional to the number of specimens carrying the same haplotype. The small black nodes indicate unobserved haplotypes. All other nodes were coloured by populations. The number of black bars between two nodes represent nucleotide differences between two haplotypes. The mitotypes of ‘Unknown’ infection status were collected from Genbank and EMBL (Additional file 1: Table S3)

Additionally, we built two types of mitochondrial haplotype networks: (A) one based on the *COI* 5′-end region only (598 bp), and (B) a second based on all four mitochondrial regions (2375 bp). The networks were built using POPART [65] with the median joining method [66] (Fig. 3). To the mitochondrial sequences produced by the present study, we added mitochondrial sequences from the same markers from any species of the *I. elegans* clade (i.e. *I. elegans*, *I. genei*, *I. saharensis*, *I. graellsii* and *I. fountaineae*), publicly available in GenBank before July 2020 (Additional file 1: Table S3). Note: only sequences with a length equal or longer to 600 bp were included to ensure the performance of the analyses.

### *Wolbachia* selective sweep

The estimation of the timing of the *Wolbachia* sweep in *I. elegans* was carried out following the method described by Rich et al. [67]. We first estimated the neutral mutation rates at the third position of fourfold and twofold synonymous codons of the four mitochondrial genes separately. The open reading frames of mitochondrial genes were found by blasting the nucleotide sequence against the mitochondrial proteome of *I. elegans* [68]. The mitochondrial sequences of *I. elegans* and *I. pumilio* were aligned in MEGA X [64] in order to calculate the number of nucleotide differences and the number of fourfold and twofold synonymous sites between the two species. Jukes–Cantor correction [69] was applied to correct for multiple substitutions. Lastly, the neutral mutation rates were calculated based on the divergence time between *I. elegans* and *I. pumilio*, estimated between 10.4 and 21.7 My before present [70] (calculations were repeated twice, using each extreme of that divergence time range). Consequently, the age of the infection can be estimated using the following equation:

1 $$t=\frac{S}{{\mu }_{a}\Sigma {n}_{i}{l}_{i}+{\mu }_{b}\Sigma {n}_{i}{m}_{i}}$$

where *t* is the estimated time since the infection; *S* is the number of observed neutral polymorphisms in a set of mitochondrial haplotypes; ${\mu }_{a}$ and ${\mu }_{b}$ are the neutral mutation rates at the third position of fourfold and twofold synonymous codons, respectively; ${n}_{i}$ is the number of sampled sequences at the ith locus; ${l}_{i}$ and ${m}_{i}$ are the number of fourfold and twofold synonymous sites at the ith locus. Our estimation was only based on mitochondrial haplotypes that were carried by the infected individuals. With this method, the corrected number of substitutions was estimated as 137.91 = 245(− 3/4)ln[1 −  (4/3) × (97/245)] and 111.11 = 332(− 1/2)ln[1 − 2 × (81/332)] among fourfold and twofold degenerate codons, respectively. Assuming the maximum estimate divergence time at 21.7 Mya [70], we estimated that neutral mutation rates on fourfold and twofold synonymous sites would be expected to be 1.30% and 0.77% per site per million years, respectively. If the minimum estimate of 10.4 Mya was assumed, the neutral mutation rates are estimated to be 2.71% and 1.61%, respectively. These estimations are biologically reasonable if we assume the general divergence rate of mtDNA in arthropods at 1.1–1.2% per site per million years (Brower 1994).

Finally, to test whether the mitochondrial genes from *Wolbachia* infected and uninfected damselflies have evolved under neutrality, we performed neutrality tests in DnaSP v6.0 [53] by calculating two population genetic statistics: Tajima’s *D* (Tajima, 1989) and Fu and Li’s *F* [54], and by estimating nucleotide diversity (*π*) and haplotype diversity (*Hd*).

### *Ischnura elegans* colour polymorphism, sex differences, and *Wolbachia* infection in Sweden

All statistical analyses were performed in R version 3.6.1 [71]. We used *Chi-*square test to investigate the association between *Wolbachia* infection and sex, and between infection and colour morph in female *I. elegans*. As most populations had a limited and incomplete sampling per sex and colour morph (e.g. only one female from Åland and from Finland mainland; only two morphs present in the Japan, Cyprus and Scotland populations, see Zenodo open data submission 10.5281/zenodo.4445061), this test was only performed using the Swedish specimens. Fisher’s exact test was applied as an improvement of *Chi*-square test when the expected value of any cells of the contingency table is below five (Table 3).

**Table 3:** Sexual and colour polymorphism in our *Wolbachia* infected and uninfected specimens from Sweden

	Female	Male	A (blue)	I (green)	O (red)
Infected (N)	72	14	27	22	23
Uninfected (N)	1	1	0	0	1

*A* androchrome, *I* infuscans, *O* obsolete

1. Infection ~ sex, ***p = 0.31***, not significant

2. Infection ~ three morphs (A, I, O), ***p = 0.63***, not significant

## Results

### *Wolbachia* penetrance and prevalence in *I. elegans* and closely related species

There were geographical variations in *Wolbachia* penetrance across the *I. elegans* populations (Table 1; Fig. 1). In Europe, our data revealed that *Wolbachia* infection was more prevalent in the north-western than in the south-eastern regions (Fig. 1). The infection frequency was over 50% in ten populations, including Sweden [SW], Scotland [AB], the Netherlands and Belgium [VW], Finland mainland [FL], Northern Japan [IK], France [FR], Italy (NI, MI, SI), and Ukraine [VC] (Note: the sample size for the latter three countries being < 5, our data might represent under or over-estimates of the *Wolbachia* infection rates in these countries). In Sweden, where the majority of our specimens came from, the infection was nearly fixed, with a frequency of 97.7% (85 individuals infected out of 87 samples tested). In contrast, all specimens from Montenegro [BJ], Greece [GR], Cyprus [CY], Finland Åland [ÅL], Ukraine (DR, PC) and Central Japan [RK] were *Wolbachia*-free. Note that the infection screening of the Japanese samples was done using leg tissues, which do not always host *Wolbachia* cells. Although all samples from [IK] were found infected, the *Wolbachia*-free status of [RK] could still be an underestimation of the true infection rate in this population. Additionally, all 13 *I. pumilio* specimens from Sweden were infected. The *I. genei* specimen from Corsica Island [CO] was infected, while the other two from Sicily [SI] and Sardinia [SD] were not. The single *I. saharensis* specimen from Morocco was also uninfected.

### *Wolbachia* strain diversity in *Ischnura elegans*,* I. genei* and *I. saharensis*

In total, three *Wolbachia* strains from the B-supergroup *Wolbachia* commonly found in insects, were identified in *I. elegans* (Table 1). They were denoted *w*Ele1, *w*Ele2, and *w*Ele3. The strain *w*Ele1 was widespread and found in Sweden [SW], Scotland [AB], Belgium and the Netherlands [VW], France [FR], Italy (MI, SI) and Ukraine [VC] (Fig. 1). In contrast, the strain *w*Ele2 was restricted to Italy (NI, MI), and *w*Ele3 was found in mainland Finland [FL] and Japan [IK]. The *I. genei* specimen from Corsica was also infected with the strain *w*Ele1; while *I. pumilio* was found to carry two divergent strains, denoted *w*Pum1 and *w*Pum2, with eight specimens (out of 13) potentially carrying the two strains simultaneously. Note that we characterized these strains using only two markers, which combination should allow the differentiation of most strains, but possibly not of all strain variants [72]. Raw reads from the *I. elegans* whole genome sequencing project [56] were assigned to *Wolbachia*, and were used to build a de novo* Wolbachia* assembly of strain *w*Ele, consisting of under 900 scaffolds. This fragmented *Wolbachia* assembly is 1.4 Mb long, suggesting it represents the full genome of a *Wolbachia* strain [73]. Although the circular genome assembly could not be built, we are confident these scaffolds are from a single bacterial infection that are not inserted in the genome of the host, as each scaffold did not contain any host genomic material.

The *Wolbachia* phylogeny based on the *ftsZ* and *wsp* genes (Fig. 2a) showed that all strains characterized in this study were from the B-supergroups (genetic distance between *w*Pip and all five strains ranged from 0.077 to 0.113). The three strains isolated from *I. elegans* formed a monophyletic group, divergent from the two strains from *I. pumilio*, *w*Pum1 and *w*Pum2. The pairwise genetic distance ranged from 1.06e^−3^ to 2.04e^−2^ among the three *I. elegans* strains, and from 3.24e^−2^ to 7.49e^−2^ between *w*Ele and *w*Pum strains (Additional file 1: Table S1). When compared to the previous strain records from the *Wolbachia* PubMLST database [55], almost all strains carried the *ftsZ* allele #7, except the strain *w*Pum2, which carried the *ftsZ* allele #73 (only one nucleotide difference from allele #7). The *wsp* allele #61 was characterized from *w*Pum2, while the four other *wsp* alleles were new to the *Wolbachia* PubMLST database, for a total of 75 polymorphic sites.

### Genetic diversity and *Wolbachia-*induced selective sweep in *I. elegans*

To identify effects of *w*Ele1 infection on host genetic diversity, we identified 25 polymorphic sites over the analysed 400 bp of the nuclear locus *PMRT* sequenced from five *I. elegans* populations. We found 1–10 genotype(s) per population, with individual genotypes showing no less than 98.1% similarity. The haplotype diversity (*Hd*) at *PMRT* was 0.88 for the *Wolbachia*-infected specimens but higher (*Hd* = 0.91) for the uninfected specimens (Table 2). Additionally, observed heterozygosity levels at two informative polymorphic sites within the *PMRT* gene (position n = 47 and 228) were similar between infected and uninfected specimens: 25% and 50% for infected vs 23.5% and 43% for uninfected specimens (two-tailed *P* = 0.86; and *P* = 0.61, respectively).

Based on the analysis of the *I. elegans COI* locus only, we detected a total of 24 mitochondrial haplotypes (Haplo1 to 24) from 169 specimens (Fig. 3a, c). The combined analysis of all four mitochondrial markers revealed additional mitotypes (17 mitotypes, Haplo_A to Q), despite all four markers being screened in 47 *I. elegans* specimens only (Fig. 3b, d). Both mitochondrial haplotype network analyses (based on *COI* and *COI* + *COIb* + *COII* + *NDI*, respectively), however, showed similar patterns, with three main distinct clades emerging (Clade 1: Japan; Clade 2: Cyprus; and Clade 3: all other populations dominated by one common mitotype, the Haplo1 or Haplo_A; Fig. 3). Note that three haplotypes, Haplo23 from Italy, Haplo24 from Japan and Haplo19 from Cyprus, characterized with the *COI* gene only, fell outside the major clades of the tree. A BLAST search for these three haplotypes in the Barcode of Life Data system (BOLD v.4, [74]) showed that Haplo19 grouped with an unidentified *Ischnura* species from Iraq (99.5% similarity to an ‘early-released’ BOLD sample—Data not provided), while Haplo24 and Haplo23 grouped with *I. elegans* specimens from Pakistan (99.85% & 99.63% similarity to BOLD accession #MAODO254-11 & #MAODO255-11, respectively). The divergence may indicate that these three mitotypes represent either polymorphism in the *COI* gene, or species misidentification. By precaution, we removed the specimens from the rest of the analyses.

Most *w*Ele1-infected specimens shared a single mitochondrial haplotype (Haplo1 or Haplo_A). However, the analysis of the four mitochondrial genes revealed that few *w*Ele1-infected specimens alternatively carry one of three closely related mitotypes (Haplo_B, Haplo_C or Haplo_F, Fig. 3b, d), all three of which show little divergence from Haplo_A. Across the geographical range of *I. elegans*, the low mitochondrial haplotype diversity among *w*Ele1-infected individuals (*Hd* = 0.13) contrasts with the high haplotype diversity found among the uninfected individuals (*Hd* = 0.70; when only *COI* is considered, Table 2). The *Wolbachia-*free Cyprus [CY] population maintained the largest mitochondrial haplotype diversity in our samples, with 11 haplotypes for 20 samples sequenced (Haplo1, Haplo13 to 22, *Ho* = 0.55). Altogether, these results are indicative of a *w*Ele1-induced selective sweep in *I. elegans,* which is also supported by significantly negative results from the neutrality tests (Tajima’s D, Fu and Li’s F; Table 2). The other two *Wolbachia* strains characterized from *I. elegans* (*w*Ele2 and *w*Ele3) were also associated with few mitochondrial haplotypes, but these haplotypes were divergent from Haplo1. The *Wolbachia* strain *w*Ele2 was associated with Haplo7 (Haplo_D and E) in Italy, while *w*Ele3 was associated to Haplo24 (Haplo_P and Q) in Japan or Haplo9 in mainland Finland (Fig. 3). However, the effects of the *Wolbachia* strains *w*Ele2 and *w*Ele3 on mitochondrial haplotype diversity remain unclear, due to our small sample size. The Haplo1 commonly found in *w*Ele1-infected *I. elegans*, was also characterized in the *w*Elel1-infected *I. genei* sample from Corsica, and the uninfected *I. saharensis* sample from Morocco. This observation could suggest hybridization between these species or, alternatively, shared infection pre-speciation of these taxa. In contrast, the *w*Pum-infected Swedish *I. pumilio* specimens carried Haplo4, and the three uninfected *I. genei* specimens from Italy carried either Haplo9 or Haplo10 (Fig. 3).

Based on the analysis of the sequences of the four mitochondrial loci, there were nine amino acid differences between the mitochondrial sequences of *I. pumilio* and *I. elegans*. Among the unchanged amino acid sites, we found 97 and 81 synonymous nucleotide differences among the 245 fourfold and 332 twofold degenerate codons, respectively. To correct for multiple substitutions and other mechanisms that cause higher number of substitutions more than the observed, we used the Jukes-Cantor correction. Given that we found four observed polymorphic sites in *w*Ele1-infected individuals, we estimated the selective sweep of wEle1 to have happened between 20,860 and 43,543 years before present. When the timing was evaluated based on the mitochondrial *COI* locus only (598 bp), which was sequenced in more samples, the sweep was estimated between 10,159 and 21,174 years before present.

### *Ischnura elegans* colour polymorphism, sex differences, and *Wolbachia* infection in Sweden

Within Sweden only, the area where most of our samples come from, we found that the three female colour morphs show similar *Wolbachia* infection frequencies (Fisher’s exact test, *p* = 0.63, Table 3) (Infection frequency: (A: blue) 1 = 27/27; (I: green) 1 = 22/22; (O: red) 0.96 = 23/24). Similarly, females and males were equally likely to be infected (Fisher’s exact test, *p* = 0.31) (Females: 0.99% or 72/73; Males: 0.93% or 14/15).

## Discussion

The spread of maternally inherited symbionts is predicted to primarily affect the mitochondrial haplotype diversity of its host [12, 75], while external ecological and demographic factors like population range expansions and bottlenecks would be expected to reduce genetic diversity at both mitochondrial and nuclear levels. We found five B-supergroup *Wolbachia* strains in the three damselfly species of the genus *Ischnura* that we investigated. The common strain *w*Ele1 was characterized in *I. elegans* and *I. genei,* while two additional strains (*w*Ele2 and 3) were found in *I. elegans* only, and two divergent strains were found in the sympatric species *I. pumilio* (*w*Pum1 and 2), which is sympatric with *I. elegans* in Sweden. In accord with a selective sweep driven by *w*Ele1 in *I. elegans* across Western Europe, we would particularly like to highlight (I) the reduced mitochondrial haplotype diversity and non-neutral evolution of the mitochondrial haplotypes associated to *w*Ele1-infected specimens, (II) the conserved nuclear haplotype diversity and levels of heterozygosity between infected and uninfected specimens. We estimated this selective sweep of *Wolbachia* to have occurred between 10,159 and 43,543 years ago, which is recent in the history of the host species (*I. elegans* and *I. graellsii* diverged 0.14 Mya [70]), but ancient enough to allow for the emergence of some diversity in the host mitochondrial DNA (Fig. 3), and largely concordant with the timing of the last glacial maximum (20,000 ya) in Europe [76]. Like other species of insects, *I. elegans* is currently shifting its geographic range northward in response to climate change [1, 33, 77, 78]. The timing of the selective sweep could suggest that *I. elegans* acquired the *w*Ele1 during its ongoing northward range expansion since the last glacial period. In such situation, one would expect that the mitochondrial diversity would be affected by both the sweep, bottlenecks and drift due to range shift, while the nuclear diversity would be only affected by bottlenecks due to range shift. Although our Swedish population show the lowest mitochondrial diversity of all populations, the nuclear diversity is high, consistent with previous data showing high genetic diversity and lack of bottlenecking during the range expansion of *I. elegans* [1]. Our sample size is however small, and more comprehensive investigations of this may inform about the consequences of the spread of *Wolbachia* for the genetic diversity, the long-term success and the dynamics of this range expanding damselfly species.

Interactions between hosts and facultative symbionts are driven by complex sets of conflicts of interests between the partners, with outcomes that also depend on the environment [79, 80]. These dynamic systems will then either result in the fixation of the symbiont in the populations [81, 82], its decline [83, 84], or stability at intermediate frequencies [85, 86]. The striking success of *w*Ele1 across the North-Western European range of *I. elegans* might suggest some benefits to the infected over the uninfected damselflies. *Ischnura elegans* is affected by parasitic water mites in nature, and previous studies have shown that tolerance and/or resistance levels to this parasite differ between sexes and female colour morphs, with for example the infuscans-obsoleta female morph showing the highest mite load when parasitized [41]. As *Wolbachia* is known to affect its host fitness in the presence of parasites [21, 22, 87], we tested whether the wEle1 infection rates varied between female colour morphs and sexes, which could then be linked to fitness variation for example in response to parasites in this host species. We found no differences in the *w*Ele1-infection frequencies between sex or morph in *I. elegans*, but this result does of course not rule out the possibility that the symbiont could still protect against parasitism in *I. elegans*. *Wolbachia* are also known to manipulate their hosts in various other ways that may similarly support its success and spread in the host populations [9, 17, 88, 89]. The strain *w*Ele1 is unlikely to manipulate its host reproductive system via feminization or male killing, as both females and males were found infected and the population sex-ratios were not systematically female biased [38, 41]. The strain could however induce cytoplasmic incompatibility, a type of sperm-egg incompatibility between males and females of different infection status [90], which would not affect population sex-ratio. Future mesocosm studies and mating experiments in semi-natural conditions [40] would allow the investigation of these hypotheses, to reveal the costs and/or benefits of *Wolbachia* in the damselfly species.

Although we found *w*Ele1 at almost fixed frequencies in the Western European populations of *I. elegans*, the strain was rare in Eastern Europe (i.e. Greece [GR], Ukraine (DR, PC), and Montenegro [BJ]), and absent from a few other populations (i.e. Cyprus [CY], Italy (NI, MI), Åland [ÅL]). We showed that the few uninfected individuals found in the Western European populations carry the same mitochondrial haplotypes as their *w*Ele1-infected conspecifics (Haplo1 and Haplo3), contrasting with the uninfected specimens from Eastern Europe that show a wide diversity of divergent mitochondrial haplotypes. This may suggest that the maternal transmission of the strain is not perfect and that few offspring from infected mothers can hatch uninfected in these populations. Additionally, two hypotheses could explain differences between populations: (I) *w*Ele1 has spread across the western populations but did not yet invade the remaining populations, or (II) *w*Ele1 has spread across the whole Europe in the past, but the infection was consequently lost in Eastern populations. For the first hypothesis, *Wolbachia*-infected population would show reduced mitochondrial haplotype diversity, while uninfected populations would have high mitochondrial diversity [10, 91]. In contrast, the second hypothesis suggests reduced haplotype diversity at the mitochondrial level and shared mitochondrial haplotypes, or closely related haplotypes, in both infected and uninfected populations [12, 88]. There is some support for each of these hypotheses, as we discuss more below.

The two populations originating from the Cyprus island in the Mediterranean Sea and from the Åland islands in the Baltic Sea, were both *Wolbachia*-uninfected. The Cyprus population is located at the southern range limit of *I. elegans* in Europe. There, the strong divergence of the mitochondrial haplotypes to those associated to the infection in Western Europe suggests that the population has remained uninfected potentially due to its geographic isolation. The population differ phenotypically in several aspects from other continental populations, particularly in terms of smaller average body size and deviant colour morph frequencies [92]. In contrast, although also geographically isolated, the island population on the Åland archipelago carries one unique haplotype that only differs by one nucleotide from the most common haplotype associated with *w*Ele1 in Sweden, and is identical to the haplotype associated with *w*Ele3 in mainland Finland (although based on one unique mitochondrial gene). These results better support the second hypothesis described above, in which the Åland population may have lost its infection recently. However, the *I. elegans* specimens collected in Åland all came from a single collection site in the southern part of the main island (i.e. Nåtö). Thus, these specimens might not be representative of the true average infection status of the entire Åland populations, and more mitochondrial genetic data will be needed to infer whether *I. elegans* colonized the Åland islands (I) after the sweep of *w*Ele1 in Sweden, or (II) after a sweep of *w*Ele3 in Finland and subsequently lost the infection and diverged at the mitochondrial level. Similarly, the uninfected individuals from Greece and Ukraine carry similar, to identical, mitochondrial haplotypes to the *w*Elel1-infected samples, which is also consistent with recent infection loss and divergence from an original population.

As a facultative symbiont, *Wolbachia* is frequently lost [83, 93], either due to drift following the colonisation of new habitats [94] or selective pressures on the symbiont. High temperatures can also negatively affect *Wolbachia* titers in some *Drosophila* species [95], and lead to the loss of the infection in mosquitoes [96] and mites [97] reared under laboratory conditions. Additionally, local population variations can also be explained by imperfect transmission of the symbiont through generations, or/and locally variable negative selective pressures on the infected individuals. This is the case for example in the Åland population of the parasitoid wasp *Hyposoter horticola* (Hymenoptera) [87], which show strong variation in *Wolbachia* penetrance across local populations (from 0 to 100% infection rate, [86]). Infection patterns across the range of *I. elegans* could thus reflect a dynamic population history with local variations in infection status and penetrance.

Our study suggests a history of short-time separation between the Italian populations, and of longer-time separation between the Japanese and European populations. In the Italian peninsula, *I. elegans* damselflies carry two closely related mitochondria (Haplo1 and Haplo6), found in association with two closely related *Wolbachia* strains (*w*Ele1 and *w*Ele2, respectively). Both mitotypes and *Wolbachia* strains may have diverged after isolation in geographically separated refugia on the Italian peninsula during the last glacial maximum [76, 98]. In contrast, *w*Ele3, the third strain characterized in all *I. elegans* specimens collected from Japan, and from mainland Finland, is highly divergent from both *w*Ele1 and *w*Ele2. The strain *w*Ele3 is found in association with two mitochondrial haplotypes (Haplo9 and Haplo24), suggesting either haplotype diversity across the large range of this particular infection, or yet undetected bacterial diversity across the large geographical distance separating Japan from Finland. Additional sampling, screening and genotyping efforts are likely to uncover a higher strain diversity in the genus *Ischnura* than already suggested here and by previous studies (in *I. senegalensis*: [47], in *I. taitensis*: [93]).

Although a maternally inherited symbiont, *Wolbachia* has been shown to also transfer horizontally between species. Examples include, but are not restricted to, the horizontal transfer of *Wolbachia* between damselfly species of the genera *Nesobasis* and *Melanesobasis* in Fiji Islands in the Pacific Ocean [48]. *Wolbachia* can be horizontally transmitted via different means, including hybridization between host species [48], shared resources (e.g. shared hostplant [99]), or shared parasitism (e.g. shared mites [100] or parasitoids [101]). Damselflies are well-known for carrying and sharing ectoparasitic water mites [41, 102], however the role of such parasites as vectors of *Wolbachia* among *Ischnura* species remains unknown. In contrast, evidences of frequent hybridization and introgression have been shown in some odonate species due to latitudinal range expansion and the increasing sympatric interactions between closely related species [103], including in the genus *Ischnura* [34–36, 104]. The fact that the same mitochondrial haplotype and the same Wolbachia strain were isolated from both *I. elegans* and *I. genei* could suggest that horizontal transfer of the strain *w*Ele1 between the two species occurred through hybridization.

## Conclusion

The present biogeographic study of *Wolbachia* in the damselfly genus *Ischnura* revealed a wide diversity of previously hidden inherited symbiotic *Wolbachia* strains in the three species investigated. Furthermore, we detected a recent selective sweep of the *Wolbachia* strain *w*Ele1 across the Western European populations of *I. elegans*, and we discuss the potential horizontal transfer of the strain through hybridization*.* The biogeographical pattern of the infection and the estimated timing of the sweep suggested that *w*Ele1 might have spread across *I. elegans* populations during its host’s northern expansion after the last glacial maximum. Consequently, the mitochondrial haplotype diversity in this range expanding species has been highly reduced but started to recover from the successful spread of the symbiont. We found the symbiont in specimens of all colour morphs and both sexes, and thus the costs and benefits from the infections remain to be investigated. We clearly highlight the loss of host genetic diversity resulting from symbiotic associations and call for greater consideration of symbiotic infections in future research on the population genetics and ability of range expanding species in the context of climate change.

## Supplementary Information

**Additional file 1: Table S1.** Pairwise genetic distance between strains using MEGA X [91]. **Table S2.** List of the primers used in this study, and associated details. **Table S3.** GenBank accession numbers from additional sequences of the mitochondrial *COI* gene from diverse *Ischnura* species from different countries. **Figure S1.** The phylogeny of (A) *ftsz* gene and (B) *wsp* gene, separately

**Additional file 2.** The wEle1 draft genome assembly (Nscaffold = 893; N50 = 5523 bp; longest scaffold = 53,331 bp,genome size = 1.4 MB) as a fasta file.

## Data Availability

The dataset supporting the conclusions of this article is available in the Zenodo repository 10.5281/zenodo.4445061; and in GenBank database (Accession #MZ463094-100; MZ501175-205; MZ508997-9001; MW509059-66; MZ893225-MZ893331) as stated in the text. The Illumina raw reads are deposited at NCBI short read archive (SRA) under accession numbers Bioproject PRJNA575663 and Biosample SAMN12906381 and SAMN12920919–20.

## Abbreviations

AB: Aberdeen population, Scotland
AI: Ain Isker population in Morocco
ÅL: Åland population in Finland
BJ: Bojana population in Montenegro
CO: Corsica population in France
*COI*: *Cytochrome c oxidase subunit 1* Coding gene, mitochondrial gene
CY: Cyprus population
DR: Dniest River population in Ukraine
*FbpA*: *Fructose-bisphosphate aldolase protein* Coding gene in *Wolbachia*
FL: Finland population
FR: France population
FtsZ: *Cell division protein* Gene in *Wolbachia*
GR: Greece population
Haplo: Mitochondrial haplotype
Hd: Haplotype diversity, as the probability of two haplotypes to be different
Ho: Observed haplotype diversity (N_H_/N_t_)
IK: Ikeda population in Japan
MI: Central Italy population
MLST: MultiLocus Sequence Typing
Na: Non applicable
N_H_: Number of haplotypes
NI: North Italy population
Nt: Number of samples
PC: Pelican City population in Ukraine
*π* (*Pi*): Nucleotide diversity
*PMRT*: *arginine methyltransferase* Nuclear gene
RK: Rokkasho population in Japan
*S*: Number of polymorphic sites
SC: Sicily population in Italy
SD: Sardinia population in Italy
SI: South Italy population
SW: Sweden population
VC: Vostochne population in Ukraine
VW: Vinne-Walem population in the Netherlands/Belgium
*w*Ele1, *w*Ele2, and *w*Ele3: *Wolbachia* strains characterized from the damselfly species *Ischnura elegans*
*w*Pum1, and *w*Pum2: *Wolbachia* strains characterized from the damselfly species *Ischnura pumilio*
*WSP*: *Wolbachia surface protein* gene
*ya*: Years ago
Mya: Million years ago
