# Supplementary material for: Wolbachia-driven selective sweep in a range expanding insect species
Source: BMC Ecol Evol. 2021 Sep 25;21:181. doi: 10.1186/s12862-021-01906-6 (PMC8466699; doi:10.1186/s12862-021-01906-6)
Supplement: Supplementary file 1 — Additional file 1: Table S1. Pairwise genetic distance between strains using MEGA X [91]. Table S2. List of the primers used in this study, and associated details. Table S3. GenBank accession numbers from additional sequences of the mitochondrial COI gene from diverse Ischnura species from different countries. Figure S1. The phylogeny of (A) ftsz gene and (B) wsp gene, separately [file 12862_2021_1906_MOESM1_ESM.pdf]

Additional materials

Table S1: Pairwise genetic distance between strains using MEGA X [91]. Each strain is characterized by two *Wolbachia* genes: *ftsZ* and *wsp*. Values in light grey are above 0.12 and differentiate A- from B-supergroup strains; values in grey are between 0.16 and 0.19 and differentiate the F-supergroup strain from all other strains; values in dark grey are above 0.19 and differentiate the D-supergroup strain from all other strains.

	1. wMel_A	2	3	4	5	6	7	8	9
2. *w*Ri_A	0,071								
3. *w*Pum2_B	0,171	0,158							
4. *w*Pum1_B	0,161	0,146	0,053						
5. *w*Ele3_B (Japan)	0,157	0,140	0,068	0,032					
6. *w*Ele2_B (Italy)	0,165	0,144	0,075	0,040	0,020				
7. *w*Ele1_B (Sweden)	0,164	0,144	0,074	0,039	0,019	0,001			
8. wPip_B	0,141	0,123	0,107	0,089	0,086	0,090	0,089		
9. *w*Bm_D	0,209	0,200	0,206	0,203	0,203	0,209	0,210	0,199	
10. *w*Clec_F	0,176	0,169	0,192	0,196	0,185	0,188	0,188	0,180	0,177

Table S2: List of the primers used in this study, and associated details.

Chromosome	Primer pair	T°C	Target	Size (bp)	Reference	Sequences (5'-3')
Wolbachia	ftsZ-F /ftsZ-R	54	Cell division protein (*ftsZ*)	435	Baldo et al. 2006 [55]	ATYATGGARCATATAAARGATA/ TCRAGYAATGGATTRGATA
Wolbachia	81F /691R	55	*Wolbachia* surface protein (*wsp*)	506	Zhou et al. 1998 [105]	TGGTCCAATAAGTGATGAAGAAAC/ AAAAATTAAACGCTACTCCA
Wolbachia	fbpA-F /fbpA-R	59	Fructose bisphos-phate aldolase (*fbpA*)	429	Baldo et al. 2006	GCTGCTCCRCTTGGYWTGAT/ CCRCCAGARAAAAYYACTATTC
Mitochondria	LCO /HCO	49	cytochrome oxidase I (*COI*)	658	Folmer et al. 1994 [51]	GGTCAACAAATCATAAAGATATTGG/ TAAACTTCAGGGTGACCAAAAAATCA
Mitochondria	C1J-2183 /TL2N-3014	49	cytochrome oxidase I (*COIb*)	811	Simon et al. 1994 [106]	CAACATTTATTTTGGTTTTTTGG/ TCCATTGCACTAATCTGCCATATTA
Mitochondria	TL2-J-3037 /C2-N-3494	57	cytochrome oxidase II (*COIIa*)	457	Simon et al. 1994	ATGGCAGATTAGTGCAATGG/ GGTAAAACTACTCGATTATCAAC
Mitochondria	IND-12586 /LRN-13398	57	NADH Deshydro-genase sub.I (*NDI*)	800	Simon et al. 1994	GTCCCTTACGAATTTGAATATAACC/ CGCCTGTTTAACAAAAACAT
Nuclear	ARG_F2 /ARG_R3	54	Arginine-methyl-transferase (*PRMT*)	512	Ferreira et al. 2014 [107]	TGCCGCCAAGGCTGGAGCATC/ TGCCACCTTCCTAATAGAGCTC

2

Table S3: GenBank accession numbers from additional sequences of the mitochondrial *COI* gene from diverse *Ischnura* species from different countries.

Accession number on GenBank	Country	Species
MH449999	Indonesia	*I. senegalensis*
KF369416	Malaysia	*I. senegalensis*
MH450003	Mauritania	*I. saharensis*
MF458738	France	*I. elegans*
MH449993	Georgia	*I. elegans*
MH449982	Belgium	*I. elegans*
KY127434	Cyprus	*I. elegans*
KY127433	Cyprus	*I. elegans*
KY127432	Cyprus	*I. elegans*
KY127437	Cyprus	*I. elegans*
KY127439	Cyprus	*I. elegans*
KY127440	Cyprus	*I. elegans*
HQ563104	Germany	*I. elegans*
KF369415	Netherlands	*I. elegans*
HM376192	Germany	*I. elegans*
KU958378	Germany	*I. elegans*
NC_031824	Germany	*I. elegans*
MK951668	China	*I. elegans*
MT216287	Poland	*I. elegans*
MT216289	Poland	*I. elegans*
MT216286	Poland	*I. elegans*
MT216295	Poland	*I. elegans*
MT216291	Poland	*I. elegans*
MT216294	Poland	*I. elegans*
MT216288	Poland	*I. elegans*
MT216292	Poland	*I. elegans*
MT216300	Poland	*I. elegans*
MT216298	Poland	*I. elegans*

MT216297	Poland	*I. elegans*
MT216290	Poland	*I. elegans*
MT216293	Poland	*I. elegans*
MT216296	Poland	*I. elegans*
MT216299	Poland	*I. elegans*

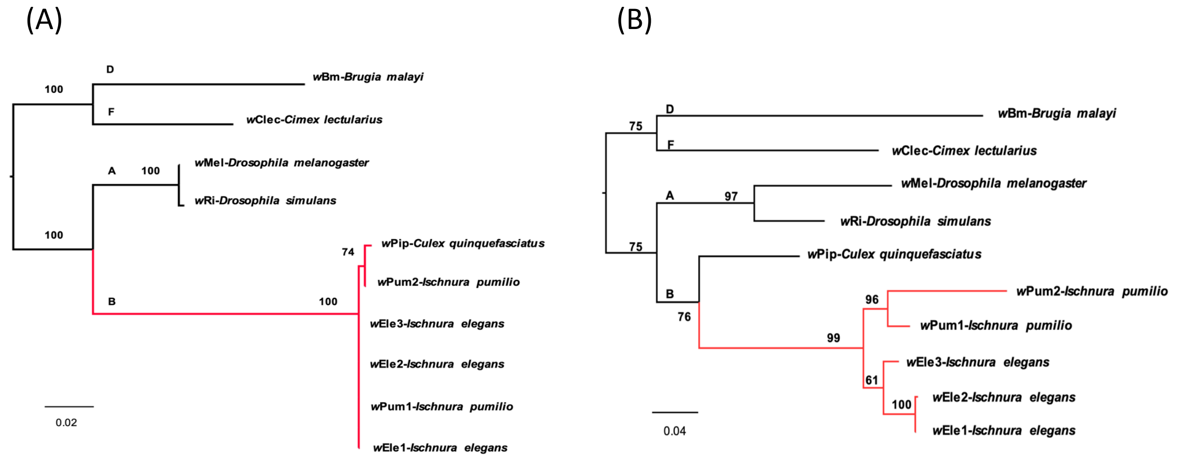

Figure S1: The phylogeny of (A) *ftsz* gene and (B) *wsp* gene, separately. Five additional strains (*w*Bm, *w*Clec, *w*Pip, *w*Mel, and *w*Ri) were included in this tree as references for the D-, F-, B- and A-supergroups, respectively. Bootstrapping was conducted using 'Ultrafast' bootstrap method in IQ-Tree with 1000 replicates. Tree was rooted using the D- and F-supergroup *Wolbachia* strains *w*Bm and *w*Clec as outgroup. The concatenated tree (Figure 2) shows a similar topology than the trees based on each gene separately, suggesting the lack of recombination between strains at these loci in this system.

Abstract translation in Chinese

演化进程中时常出现一些空间上的遗传结构。这些遗传结构可表现为区域性遗传多样性的丧失，或是线粒体和细胞核基因在演化历史上的不匹配。而对这些遗传结构的研究能够帮助我们确定其背后隐藏的机制。沃尔巴克氏菌（*Wolbachia*）作为一类经由宿主母系遗传的细菌，被认为是昆虫中感染最广泛的共生体（symbiont）。沃尔巴克氏菌时常通过直接提升宿主的适合度，或改变宿主的繁殖模式使得受感染的雌性个体拥有比未感染个体更高的适合度，来达到在宿主种群中快速传播的目的。在足够的选择压力下，优势共生菌株在种群中的频率将逐渐上升，而和其相关联的宿主线粒体基因型的频率也将随之增加（i.e. hitchhike），最终表现为宿主线粒体遗传多样性的降低。近几年，长叶异痣蟌（*Ischnura elegans*: van der Linden, 1820）逐渐的作为模式物种，用于研究气候变化下物种生境扩张对遗传结构的影响。尽管越来越多的研究在长叶异痣蟌种群中证实了此类影响，对异痣蟌属沃尔巴克氏菌的研究却是一片空白。在这项研究中，我们将展示沃尔巴克氏菌在17个分布于欧洲和日本的长叶异痣蟌种群以及部分来自地中海地区的近邻物种（i.e. *I. genei*: Rambur, 1842; *I. saharensis*: Aguesse, 1958）种群中感染率的地理变化。

这项研究 (a) 确定了多个沃尔巴克氏菌谱系的基因型，(b) 为杂交诱导的宿主间共生体传播提供证据，(c) 揭示纬度和感染率可能的相关性（i.e. 高纬度种群的受感染程度更高），以及 (d) 揭示欧洲西北部长叶异痣蟌种群线粒体遗传多样性的缺失，暗示其可能受到的共生体连锁效应（i.e. hitchhike, selective sweep）。在欧洲西北部受沃尔巴克氏菌感染的种群（瑞典，苏格兰，荷兰，比利时，法国和意大利）中，线粒体基因型的多样性呈现较低水平；相反，未受感染或感染程度较低的种群（乌克兰，希腊，黑山共和国和塞浦路斯）拥有相对较高的线粒体遗传多样性。根据现有模型估计，沃尔巴克氏菌在长叶异痣蟌种群中的传播可以追溯到约2万至4万4千年前。这一时间点和约2万年前结束的末次冰期相吻合。

我们的研究为“共生体如何在宿主的后冰期生境扩张中塑造遗传结构“提供了一个典例。这项研究也通过展示共生体在空间尺度上对宿主线粒体遗传多样性的影响，对那些没有考虑共生体的昆虫种群遗传学研究提出挑战。

Keywords: 内共生，谱系生物地理学，蟌，线粒体，遗传多样性
